# Mental and physical health, and long-term quality of life among service members injured on deployment

**DOI:** 10.1186/s12955-021-01852-3

**Published:** 2021-09-16

**Authors:** Cameron T. McCabe, Jessica R. Watrous, Susan L. Eskridge, Michael R. Galarneau

**Affiliations:** 1 Operational Readiness Directorate, Naval Health Research Center, 140 Sylvester Road, San Diego, CA 92106 USA; 2 Leidos, 140 Sylvester Road, San Diego, CA 92106 USA

**Keywords:** Health-related quality of life, Military, Extremity injury, Traumatic brain injury, PTSD, Musculoskeletal disorders, Cardiovascular disorders

## Abstract

**Background:**

More than 52,000 casualties have been documented in post-9/11 conflicts. Service members with extremity injuries (EIs) or traumatic brain injury (TBI) may be at particular risk for long-term deficits in mental and physical health functioning compared with service members with other injuries.

**Methods:**

The present study combined medical records with patient reports of mental health and health-related quality of life (HRQOL) for 2,537 service members injured in overseas contingency operations who participated in the Wounded Warrior Recovery Project. Combined parallel-serial mediation models were tested to examine the pathways through which injury is related to mental and physical health conditions, and long-term HRQOL.

**Results:**

Results revealed that injury was indirectly related to long-term HRQOL via its associations with physical health complications and mental health symptoms. Relative to TBI, EI was associated with a higher likelihood for a postinjury diagnosis for a musculoskeletal condition, which were related to lower levels of later posttraumatic stress disorder (PTSD) symptoms, and higher levels of physical and mental HRQOL. Similarly, EI was related to a lower likelihood for a postinjury PTSD diagnosis, and lower levels of subsequent PTSD symptoms, and therefore higher physical and mental HRQOL relative to those with TBI. Despite this, the prevalence of probable PTSD among those with EI was high (35%). Implications for intervention, rehabilitation, and future research are discussed.

## Introduction

More than 52,000 U.S. service members have been wounded in action during overseas contingency operations following 9/11 [1]. Extremity injuries (EIs) and traumatic brain injuries (TBIs) have been described as the characteristic injuries of recent conflicts [2, 3], comprising a majority of injuries sustained [4]. Although acute symptoms of EI (e.g., pain, loss of function) and TBI (e.g., headache, sleep disturbances, cognitive problems) are well documented [5–8], less is known about their long-term impact for health and functioning. As the sequelae and health care needs of those injured on deployment vary based on the type, mechanism, and severity of injury, it is imperative to delineate the pathways through which combat-related injury contributes to long-term health-related quality of life (HRQOL). Further, although medical care is available to service members and veterans through Department of Defense (DoD) or Veterans Affairs facilities, many are opting to seek care through community providers [9]. Thus, it is important for both military and civilian providers to understand the long-term effects of injury in order to optimize prevention and intervention efforts to meet the growing needs of wounded warriors.

### Health-related quality of life

HRQOL has been described as an individual’s perception of their mental, physical, emotional, and social well-being at a given point in time [10]. As a concept, HRQOL incorporates aspects of one’s objective medical status in concert with subjective appraisals of one’s own functioning, and represents a critical patient-reported outcome [11]. Prior research among military and civilian populations suggests traumatic injuries are associated with both mental and physical health consequences, which may influence long-term HRQOL [8, 12, 13]. TBI has frequently been linked with lower perceived HRQOL among post-9/11 and Gulf War era veterans [13, 14]. Similarly, EI has been associated with lower mental and physical health HRQOL [15]. In a longitudinal study of civilian patients with lower EIs, having experienced pain, as reported by the patient, was associated with worse health functioning. Over time, however, this association weakened, and was largely explained by changes in anxiety and depressive symptoms at 2 years postinjury [8].

### Posttraumatic stress disorder

Posttraumatic stress disorder (PTSD) is prevalent among service members who have deployed [16]. A recent meta-analysis suggests an estimated 23% of Operation Enduring Freedom and Operation Iraqi Freedom veterans are thought to have a PTSD diagnosis [17]. Higher rates may exist among those who sustained traumatic injuries [18–20], and while robust evidence exists linking TBI with PTSD and other mental health concerns [21, 22], mixed evidence exists with respect to the relationship between EI, PTSD, and HRQOL. In one study of U.S. service members who sustained an extremity vascular injury, Scott and colleagues [15] found high rates of pain and psychiatric disorders, and both depression and PTSD were shown to be associated with lower mental HRQOL. Characteristics of the injury themselves (e.g., limb salvage, secondary amputation), however, were not associated with long-term prognosis. In contrast, when accounting for other injuries, particularly injuries to the head and spine, Woodruff and colleagues [20] found little evidence that upper or lower EIs were correlated with mental health status or HRQOL.

### Physical health problems

Although some studies contend that service members may be healthier relative to the general public (see healthy warrior effect)[23], those injured in the line of duty experience a number of physical health complications, including heightened risk for musculoskeletal disorders (MSDs)[24] and cardiovascular disorders (CVDs) [25, 26]. MSDs are among the top reasons for medical discharge from the military [24] and for diminished readiness [27]. They also increase the likelihood of sustaining new injuries, may prolong recovery time from previous injuries [28], and have been linked to poor mental and physical health functioning and HRQOL [24].

Individuals with EI in particular, including severe EIs such as amputation or limb salvage, are at risk for both acute and chronic health complications that may require clinical intervention (e.g., pain, osteoarthritis, osteoporosis, or other musculoskeletal conditions) [29]. Moreover, individuals with lower extremity amputations are at higher risk of developing secondary health conditions (e.g., osteoarthritis, low back pain), which can contribute to lower HRQOL over time [30, 31].

Musculoskeletal conditions are also prevalent following TBI [32]. In their 2011 retrospective study of individuals hospitalized with a TBI, Brown and colleagues found nearly 80% of their sample reported musculoskeletal issues in the past 30 days, including pain, joint aches or stiffness an average of 26 years after injury, and individuals with musculoskeletal complaints reported greater bodily pain and physical health issues which impacted their daily activities relative to those without physical complaints. However, in contrast to EI, links between TBI and musculoskeletal complaints may be driven by the mechanism of injury itself which may cause trauma to other areas of the body [32].

By virtue of being exposed to a traumatic stressor, those wounded in combat may experience heightened stress response, activation of the hypothalamic–pituitary–adrenal axis, and chronic inflammation, which may damage the cardiovascular system and contribute to the development of CVDs over time [33, 34]. In their 2015 retrospective study of combat injury among U.S. service members, Stewart and colleagues demonstrated that higher injury severity was associated with greater risk of hypertension, diabetes mellitus, chronic kidney disease, and coronary artery disease [34]. Further, according to a population-based study examining blood pressure readings, as many as 13% of active duty service members may have current hypertension [35], and rates of self-reported hypertension have been shown to be higher among those diagnosed with PTSD [26] and among service members with severe EIs [29].

Individuals with a TBI may also be at heightened risk for chronic cardiovascular problems [36], however similar to links between TBI and MSD, this may be a function of secondary health complications experienced following TBI. Specifically, individuals with TBI experience sleep problems [37], inflammation and diminished immune response [38], which contribute to long-term cardiovascular declines. Further, Nyam and colleagues [36] showed that relative to controls, patients with TBI were at significantly higher risk for experiencing major acute cardiovascular and cerebrovascular events, cardiovascular disease, stroke, and death.

#### Study objectives

Adverse mental and physical health conditions are commonplace among service members with combat-related injuries [18, 20], and may represent mechanisms that link injury (e.g., EI, TBI) and long-term HRQOL. Although EI and TBI may be uniquely related to HRQOL outcomes, they may operate through different mechanisms and be differentially associated with secondary health complications to a greater (or lesser) extent. To date, however, this possibility has not been explored. Thus, in order to identify novel targets for prevention and intervention and optimize existing rehabilitation efforts, it is crucial to delineate potential risk factors that differentiate common injury types sustained by service members, (e.g., EI and TBI) and contribute to HRQOL deficits. Using objective medical records, in concert with data from a longitudinal examination of patient-reported outcomes of injured service members, the present study examined the pathways through which injury is related to mental and physical health conditions and long-term HRQOL. Specifically, we tested a parallel-serial mediation model whereby the associations between injury type (EI vs. TBI) and physical and mental components of HRQOL were indirect through postinjury mental (PTSD) or physical health diagnosis (MSD and CVD) and current PTSD symptomology.

We first hypothesized that relative to those with a documented TBI, service members with a documented EI (upper or lower) would be significantly less likely than those with a TBI to have a postinjury diagnosis for PTSD (H1a). In contrast, we hypothesized that individuals with an EI would be significantly more likely than those with a TBI to have a postinjury diagnosis for an MSD (H1b) and CVD (H1c). Next, because mental and physical health conditions may exacerbate existing symptoms and potentially interfere with recovery, we hypothesized that the presence of a postinjury PTSD or physical health diagnosis would be associated with higher current self-reported PTSD symptomology (H2a-H2c). Third, we examined whether postinjury PTSD (H3a, H3b) and physical health diagnoses (H3c-H3f) and current PTSD symptomology (H3g, H3h) were directly related to physical and mental HRQOL. Finally, we tested parallel-serial indirect effects of injury on physical and mental HRQOL through postinjury PTSD and physical health diagnoses and PTSD symptomology (H4a-H4f).

## Methods

### Participants

The study population consisted of participants in the Wounded Warrior Recovery Project (WWRP). The WWRP tracks the long-term, patient-reported outcomes of service members in the Expeditionary Medical Encounter Database (EMED), a U.S. Navy-maintained deployment health database [39]. Recruitment efforts began in November 2012, and continue on a rolling basis. Individuals who sustained a documented injury (minor to severe) while on deployment after December 2001 were contacted via email and/or postal mail, and consented to participate in a 15-year follow-up study. Each participant received a $20 incentive for each survey battery completed. Additional details regarding WWRP methodology are available elsewhere [40]. The current study includes a subsample of all WWRP participants who enrolled in and completed relevant assessments prior to data extraction. Participants were injured between December 2001 and August 2017, and completed a WWRP assessment between September 2018 and April 2020. Overall, 3,909 participants completed an assessment during this period, We excluded 1,170 participants who either (a) did not have a documented EI or probable TBI (n = 399) or (b) had both a documented EI and probable TBI (n = 771). An additional 202 individuals were excluded from analyses due to incomplete survey or demographic data, resulting in a final study sample of 2,537 participants.

### Measures

#### Sociodemographic and injury-related information

Descriptive and injury-related variables were gathered from EMED. EIs and TBIs were identified using the Abbreviated Injury Scale (AIS) [41]. Injury Severity Scores (ISSs) [42] were calculated from AIS codes and quantified overall trauma severity of each individual. Possible ISS values range from 0 (*no injury*) to 75 (*fatal injury*), and scores from the present study ranged from 1 to 59. In addition, we identified participant age, service branch, military grade, and injury mechanism at the time of time of injury.

### Diagnostic information

The number of medical encounters for mental and physical health conditions were calculated from inpatient and outpatient health records obtained from the Department of Defense Military Health System Data Repository using *International Classification of Diseases, Ninth and Tenth Revisions, Clinical Modification* (ICD-9-CM and ICD-10-CM, respectively) codes. Of note, medical encounters were restricted to those that occurred after the injury but prior to responding to the WWRP questionnaire. As shown in Table 1, CVDs included hypertensive, ischaemic heart disorders, heart failure, and arrhythmia, whereas codes associated with inflammatory diseases were excluded. In addition, diagnostic code groupings representing common MSDs (e.g., osteoarthritis, joint and back disorders, stress fractures) and inflammatory processes were included. Diffuse diseases of connective tissue were excluded. Finally, diagnostic codes representing PTSD were included. Participants were considered to have a given condition if they had at least one inpatient or two outpatient encounters [43, 44].

**Table 1:** *ICD-9-CM* and *ICD-10-CM* code groups

Code group	*ICD-9-CM* code	*ICD-10-CM* code
PTSD	30,981	F4310–F4312
Musculoskeletal disorders	71,500–71,598, 71,640–71,740, 71,742–71,819, 71,830–71,859, 71,880–71,919, 71,940–72,142, 7216, 7218–7249, 7260–72,701, 7295, 73,399	M1280–M1389, M150, M153, M158–M1612, M167–M1712, M174–M1812, M184–M19079, M19211–M1991, M1993, M222X1–M222X9, M2240–M2242, M23000–M23009, M23241–M23249, M23261–M2352, M238X1–M24176, M2440–M24443, M24451–M24476, M2450–M2518, M2540–M259, M4325–M436, M438X9, M450–M461, M4640–M4649, M4680–M4699, M4710–M4819, M489–M546, M5489–M549, M6580–M659, M6780–M6788, M7010–M7072, M7500–M7582, M7610–M7672, M76821–M7712, M7730–M779, M79601–M79676, M8930–M8939, M898X0–M899, M948X0–M949, R262, R29898–R2991
Cardiovascular disorders	4010–4149, 4160–4179, 4230–4293, 4295–4299, 4400–4409, 4411–4429, 44,389–4439, 4460–4489, 4580–4599, 7850–7853, 78,550–78,559, 7859	I10–I150, I158–I159, I200–I214, I230, I240–I248, I2510–I255, I25810–I259, I270–I2720, I2781–I2782, I2789–I300, I308–I319, I339, I340–I39, I420–I479,I480–I482, I484–I519, I700–I70799, I708–I7092, I7389–I739, I87001–I87399, I970–I97191, I998–I999, R000–R012, R0989, R58, R9430–R9439

ICD-9-CM = International classification of diseases, Ninth Revision, Clinical Modification; ICD-10-CM = International Classification of Diseases, Tenth Revision, Clinical Modification; PTSD = posttraumatic stress disorder

### Posttraumatic stress disorder

PTSD symptom severity was assessed using the 20-item PTSD Checklist for the DSM-5 (PCL-5)[45] a widely used and validated measure of PTSD symptoms and severity over the past month. Questions refer to concerns that may arise in response to a “very stressful” life experience, and participants rated how bothered they were by each symptom using a scale of 0 (*not at all*) to 4 (*extremely*). Sample responses include “repeated, disturbing memories, thoughts, or images of a stressful experience from the past” and “feeling jumpy or easily startled.” Scores were summed (*M* = 27.71, *SD* = 20.15; Cronbach’s ⍺ = 0.97), with higher scores representing greater symptom severity, and scores 33 or greater indicating a positive screen for PTSD [46].

### Quality of life

HRQOL was assessed using the physical (PCS) and mental health component scores (MCS) calculated from the 36-item Short Form Survey (SF-36) [47]. The SF-36 has been widely used among active duty and veteran samples [48–50]. The PCS and MCS component scores are calculated using a norm-based algorithm that allows for the calculation of two, norm-based scores Functional limitations due to (1) physical health or (2) emotional problems, energy and fatigue, emotional well-being, social functioning, pain, and general health. The PCS and MCS encapsulate physical and mental HRQOL respectively, and demonstrate good internal consistency (Cronbach’s αs ranged from 0.84 to 0.93). In August 2019, three items were removed from the WWRP assessment as part of an ongoing DoD effort to reduce undue burden on Active Duty service members participating in survey research. Consistent with scoring guidelines [51], mean imputation was used to impute item-level scores for subscales which participants responded to at least 50% of the items. Scores ranged from 14.07 to 69.80 on the PCS and 2.73 to 70.83 on the MCS, with higher scores representing better functioning (*M*_*PCS*_ = 43.44, *SD*_*PCS*_ = 10.19; *M*_*MCS*_ = 40.08, *SD*_*MCS*_ = 13.50).

### Statistical analyses

A path model with parallel and serial mediators was run using Mplus version 8.6 [52] with weighted least square mean and variance adjusted estimation (WLSMV). A fully saturated (just-identified) model was specified to test whether injury was associated with postinjury PTSD (*a*_*1*_), MSD (*a*_*2*_), and CVD diagnoses (*a*_*3*_)*,* as well as self-reported PTSD symptoms (*a*_*4*_), and whether each factor was uniquely associated with HRQOL scores (PCS or MCS; *b*_*1*_–*b*_*6*_). Further, this model specified additional indirect pathways, where injury was related to PCS and MCS scores through postinjury PTSD (*d*_*1*_), MSD (*d*_2_), and CVD encounters (*d*_3_), and their impact on self-reported PTSD symptoms (*b*_7_, *b*_8_). Adjusted indirect effects for dichotomous mediators were calculated using methods described in Stride and colleagues (2015) model 4c [53]. Bias-corrected bootstrapping with resampling was used to generate 95% confidence intervals for all direct and indirect effects. Confidence intervals that do not contain a value of zero are considered statistically different from zero.

## Results

### Descriptive statistics

Descriptive information and sample characteristics are shown in Table 2. Approximately 58% (*n* = 1,465) of study participants had an EI, and 42% had a documented TBI (*n* = 1,072). Overall, most participants were male (94.3%), enlisted (88.1%), and in the Army (68.3%) or Marine Corps (27.0%) when they were injured, and most injuries were blast related (76.0%). About 38% of participants screened positive for PTSD based on standardize cutoffs for the PCL-5.

**Table 2:** Demographic characteristics of study participants^a^

	Overall (*n* = 2,537)	EI (*n* = 1,465)	TBI (*n* = 1,072)
Age at first survey (years)			
18–24	7 (0.3)	1 (0.1)	6 (0.6)
25–29	266 (10.5)	82 (5.6)	184 (17.2)
30–39	1491 (58.8)	850 (58.0)	641 (59.8)
40–49	567 (22.3)	385 (26.3)	182 (17.0)
50 +	206 (8.1)	147 (10.0)	59 (5.5)
Sex			
Male	2392 (94.3)	1405 (95.9)	987 (92.1)
Female	145 (5.7)	60 (4.1)	85 (7.9)
Race/ethnicity			
Hispanic or Latino	288 (11.6)	158 (11.1)	130 (12.3)
Non-Hispanic, White	1916 (77.3)	1106 (77.8)	810 (76.6)
Black or African American	165 (6.7)	93 (6.5)	72 (6.8)
Other/unspecified	111 (4.5)	65 (4.6)	46 (4.3)
Marital status			
Married	1425 (56.2)	810 (55.3)	615 (57.4)
Unmarried	1112 (43.8)	655 (44.7)	457 (42.6)
Military grade			
Enlisted	2125 (88.1)	1185 (86.2)	940 (90.7)
Officer	286 (11.9)	190 (13.8)	96 (9.3)
Service branch			
Air Force	42 (1.7)	17 (1.2)	25 (2.3)
Army	1733 (68.3)	1037 (70.8)	696 (64.9)
Marine Corps	686 (27.0)	363 (24.8)	323 (30.1)
Navy	76 (3.0)	48 (3.3)	28 (2.6)
Injury mechanism			
Blast	1928 (76.0)	991 (67.6)	937 (87.4)
Gunshot wound	418 (16.5)	341 (23.3)	77 (7.2)
Other/unknown	191 (7.5)	133 (9.0)	58 (5.5)
Injury severity score	4.9 (5.8)	6.0 (6.6)	3.5 (4.0)
Time since injury (years)	10.4 (3.3)	11.6 (3.0)	8.7 (2.9)
Medical encounters^b^			
Cardiovascular disorders (%)	637 (25.1)	402 (27.4)	235 (21.9)
Musculoskeletal disorders (%)	1982 (78.1)	1219 (83.2)	763 (71.2)
PTSD (%)	839 (33.1)	457 (31.2)	382 (35.6)
Self-reported symptoms			
PCL-5 positive screening (%)	963 (38.0)	507 (34.6)	456 (42.5)
PCL-5 score	27.7 (20.2)	26.0 (19.7)	30.1 (20.6)
Physical component score	43.8 (10.2)	43.6 (10.3)	44.0 (10.0)
Mental component score	40.1 (13.5)	41.1 (13.7)	38.8 (13.1)

EI = extremity injury; PCL-5 = Posttraumatic Stress Disorder Checklist for DSM-5; PTSD = posttraumatic stress disorder; TBI = traumatic brain injury

^a^Data exclude missing values. ^b^Percentage of medical encounters reflect proportion of participants with at least one inpatient or two outpatient diagnoses for a given condition

**Table 3:** Indirect effects of injury on HRQOL through postinjury physical and mental health mediators

	*IE*	*SE*	95% BCCI
Injury → PTSD Dx → SF-36 Physical	**-0.06**	**0.02**	**[-0.09; -0.03]**
Injury → PTSD Dx → SF-36 Mental	**0.07**	**0.02**	**[0.04; 0.10]**
Injury → MSD Dx → SF-36 Physical	**-0.09**	**0.02**	**[-0.12; -0.05]**
Injury → MSD Dx → SF-36 Mental	**0.09**	**0.02**	**[0.05; 0.13]**
Injury → CVD Dx → SF-36 Physical	0.00	0.01	[-0.02; 0.02]
Injury → CVD Dx → SF-36 Mental	0.00	0.01	[-0.02; 0.02]
Injury → PTSD (WWRP) → SF-36 Physical	0.06	0.05	[-0.05; 0.14]
Injury → PTSD (WWRP) → SF-36 Mental	0.15	0.12	[-0.11; 0.32]
Injury → PTSD Dx → PTSD (WWRP) → SF-36 Physical	**0.01**	**0.00**	**[0.01; 0.02]**
Injury → PTSD Dx → PTSD (WWRP) → SF-36 Mental	**0.03**	**0.01**	**[0.02; 0.05]**
Injury → MSD Dx → PTSD (WWRP) → SF-36 Physical	**0.02**	**0.01**	**[0.01; 0.03]**
Injury → MSD Dx → PTSD (WWRP) → SF-36 Mental	**0.04**	**0.01**	**[0.02; 0.06]**
Injury → CVD Dx → PTSD (WWRP) → SF-36 Physical	0.00	0.00	[-0.00; 0.00]
Injury → CVD Dx → PTSD (WWRP) → SF-36 Mental	0.00	0.00	[-0.01; 0.01]

Effects in **bold** are significantly different from zero

BCCI = bias-corrected confidence interval; CVD = cardiovascular disorder; Dx = diagnosis; HRQOL = health-related quality of life; IE = indirect effect; MSD = musculoskeletal disorder; PTSD = posttraumatic stress disorder; SE = standard error; SF-36 = 36-item Short Form Survey; WWRP = Wounded Warrior Recovery Project

### Indirect effects

#### PTSD diagnoses

Hypothesis H1a was supported. Relative to service members with a TBI, those with an EI were significantly less likely than those with TBI to have a documented postinjury PTSD diagnosis (*b* =  − 0.24, *p* < 0.001). In addition, postinjury PTSD diagnosis was associated with significantly higher (better) PCS scores when controlling for postinjury physical health diagnoses, self-reported mental health symptoms, and study covariates (*b* = 1.15, *p* = 0.01)(H3a). A significant indirect effect was observed where injury type was related to PCS through postinjury PTSD diagnosis (*ab* = -0.06, 95% bias-corrected confidence interval [BCCI] [-0.09; -0.03]). In contrast, postinjury PTSD diagnosis was related to significantly lower MCS scores (*b* = -1.14, *p* < 0.01)(H3b), and the association between injury and MCS scores was indirect through postinjury PTSD diagnosis (*ab* = 0.07, 95% BCCI: [0.04; 0.10]).

### Physical health diagnoses

Relative to service members with a TBI, those with an EI were significantly more likely to have a documented MSD diagnosis (*b* = 0.32, *p* < 0.001), supporting hypothesis H1b. Further, MSD diagnoses were associated with significantly lower (worse) PCS scores (*b* =  − 3.39, *p* < 0.001) (H3c). In contrast, postinjury MSD diagnosis was related to significantly higher (better) MCS scores (*b* = 1.53, *p* < 0.001) (H3d). The indirect effects of injury on PCS (*ab* = -0.09, 95% BCCI: [-0.12; -0.05]) and MCS (*ab* = 0.09, 95% BCCI: [0.05; 0.13]) through postinjury MSD diagnosis were significantly different from zero (see Table 3).

When adjusting for postinjury PTSD and MSD diagnoses, and study covariates, service members with an EI were not significantly more likely than those with a TBI to have a documented CVD diagnosis (*b* = 0.00, *p* < 0.99), nor were postinjury CVD diagnoses significantly related to PCS scores (*b* =  − 0.57, *p* = 0.09) or MCS scores (*b* = 0.02, *p* = 0.95). Thus, hypotheses H1c, and H3e and H3f were not supported. No significant indirect effects were observed for injury type on PCS or MCS through postinjury CVD diagnoses.

### Self-reported PTSD symptoms

Injury type was not significantly related to level of PTSD symptoms on the WWRP assessment (*b* =  − 1.14, *p* = 0.26). The presence of a postinjury PTSD diagnosis was associated with significantly higher levels of PTSD symptoms (*b* = 11.29, *p* < 0.001)(H2a), whereas postinjury MSD diagnosis was associated with significantly lower levels of PTSD symptoms (*b* =  − 4.18, *p* < 0.001)(H2b). Contrary to our hypothesis (H2c), postinjury CVD diagnosis was not associated with self-reported PTSD symptoms (*b* = -0.07, *p* = 0.92).

**Fig. 1:**
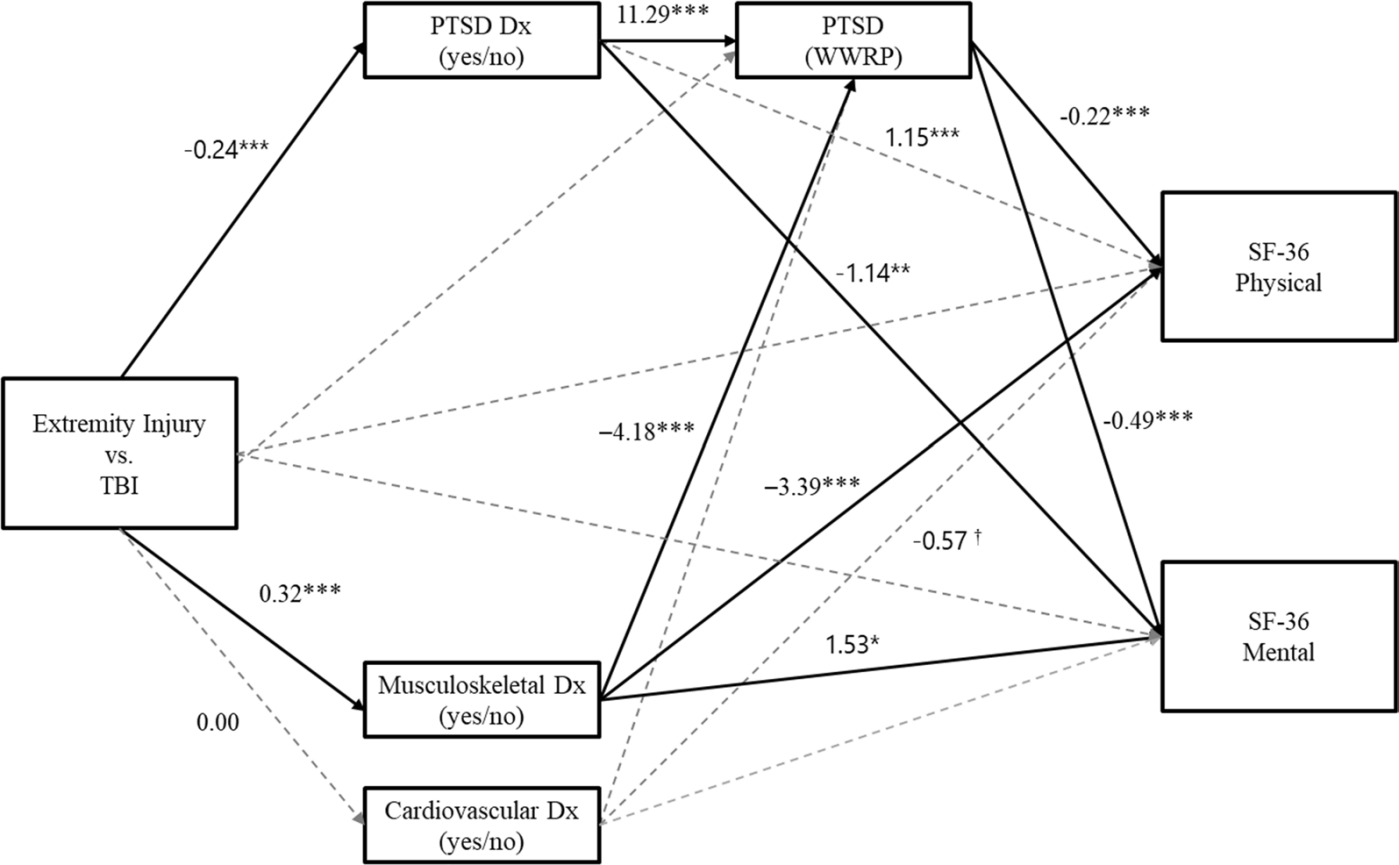
Unstandardized effects of injury on health-related quality of life through postinjury physical and mental health mediators. Solid lines indicate significant indirect effect pathways; Dx = diagnosis; PTSD = posttraumatic stress disorder; SF-36 = 36-item Short Form Survey; TBI = traumatic brain injury; WWRP = Wounded Warrior Recovery Project. ^†^*p* < .10; *p < .05; **p < .01; ***p < .001

Controlling for postinjury PTSD and physical health diagnoses and study covariates, PTSD symptoms were associated with significantly lower (worse) PCS (*b* =  − 0.22, *p* < 0.001)(H3g) and MCS scores (*b* =  − 0.49, *p* < 0.001)(H3h). As shown in Table 3, results suggest that injury type was related to both PCS and MCS through sequential associations with postinjury PTSD diagnoses and self-reported PTSD symptoms (see also Fig. 1). Specifically, relative to those with a TBI, service members with an EI had significantly higher (better) PCS and MCS scores by virtue of a lower likelihood of having a postinjury PTSD diagnosis, and therefore lower self-reported PTSD symptoms (H4a and H4b respectively).

A similar sequential indirect effect was found where injury type was related to PCS and MCS scores through postinjury MSD diagnoses and self-reported PTSD symptoms (Fig. 1). Relative to those with a TBI, service members with an EI had significantly higher (better) PCS and MCS scores by virtue of having a higher likelihood of having a postinjury MSD diagnosis and lower levels of self-reported PTSD symptoms (H4c and H4d respectively). No other direct or indirect effects were observed, thus H4e and H4f were not supported.

## Discussion

Military service is a high-risk occupation where service members may be exposed to myriad hazards that affect health functioning. Service members who are injured may be at higher risk for development of later problems, yet the type and severity of injuries may manifest in unique sequelae over time. As the long-term needs and financial costs of care for wounded service members continue to rise [54, 55], it is vital to identify the pathways through which common types of injuries (e.g., EI, TBI) relate to long-term health outcomes. By leveraging objective medical records with later self-reported symptoms, the present study elaborated on and extended prior health research among wounded service members, and identified multiple pathways through which injury can affect later HRQOL, demonstrating that EI and TBI were differentially related to long-term mental health symptoms and postinjury mental and physical health complications. Moreover, long-term associations between injury type and HRQOL were shown to be indirect via their association with one’s level of current PTSD symptoms and likelihood of having a postinjury diagnosis for PTSD, and also one’s likelihood of having a diagnosis for a musculoskeletal problem. These mechanisms represent critical targets of prevention and intervention for these injuries, and findings provide support for the importance of addressing both mental and physical health domains to promote health and improve the HRQOL of injured service members.

Results revealed that when adjusting for PTSD and physical health diagnoses, injury severity, and time elapsed since the injury, injury type was indirectly related to long-term HRQOL through a higher likelihood for having a postinjury musculoskeletal diagnosis, and a lower level of current PTSD symptoms. That is, despite those who sustained an EI being significantly more likely to have a musculoskeletal diagnosis than those with TBI, musculoskeletal diagnoses were associated with significantly *lower* PTSD symptoms, and this combination was associated with significantly higher physical and mental HRQOL. Similarly, injury type was also indirectly related to HRQOL through postinjury PTSD diagnosis and subsequent PTSD symptoms, where participants with an EI (relative to those with a TBI) reported significantly higher (better) physical and mental HRQOL through a lower likelihood of having a postinjury diagnosis for PTSD, which was associated with lower levels of PTSD symptoms.

Importantly, although we have framed these effects largely in terms of the relationship between EI and HRQOL, these effects can also be interpreted through the lens of TBI. Specifically, it is also true that TBI was associated with significantly lower likelihood of having a documented postinjury MSD diagnosis, which in turn was associated with significantly higher PTSD symptoms, and lower (worse) physical and mental HRQOL relative to those with EI. Similarly, TBI was associated with a significantly higher likelihood of having a documented postinjury PTSD diagnosis, higher subsequent PTSD symptoms, and lower (worse) physical and mental HRQOL relative to those with EI.

Results from the present study reinforce the need for optimized and integrated treatment for injured service members. As service members and veterans increase their utilization of community health resources [9], it remains critical for military and civilian providers to be aware that the mental and physical health of their patients are inextricably linked. For example, individuals with TBI in this study reported worse physical health functioning relative to those with EI despite being at lower risk for having a diagnosed physical health condition (i.e., MSD), and this effect could largely be explained through higher levels of PTSD symptoms. Further, although they did not examine mediational pathways in their epidemiological study of Australian Gulf War veterans, Kelsall and colleagues [24] demonstrated that those with MSD and comorbid PTSD or depression experience significantly lower mental and physical health functioning relative to those with MSD alone. They suggested that PTSD and/or depression may have exacerbated existing physical health symptoms or interfered with treatment effectiveness and recovery, thus resulting in worse health overall. To our knowledge, our study is one of the first studies to examine these indirect associations, and these findings emphasize the relevance of psychological health factors, in this case PTSD symptoms, for understanding pathways from injury to both mental and physical health outcomes.

We did not find evidence to suggest that injury type is indirectly related to HRQOL through cardiovascular diagnosis. That is not to say the experience of cardiovascular problems are unimportant in this process. Prior research demonstrates CVDs are a leading cause of morbidity and mortality [56], contribute to lower HRQOL [57], and may be higher among those with PTSD or depression [43, 58]. To the extent that individuals with CVD are asymptomatic, CVD may not influence how service members perceive their HRQOL. That is, individuals who are unaware of or feel largely unencumbered by CVD-related symptoms, such as high blood pressure, may be less likely to attribute difficulties in their daily functioning to CVD, particularly if they are relatively young and otherwise healthy. This could change as conditions persist and participants age, a plausible consideration given that roughly 70% of participants were less than 39 years old at the time of the study. Another possibility is that other factors such as genetic predisposition, mental health, and certain health behaviors (e.g., sedentary lifestyle, alcohol and tobacco use, poor sleep hygiene), which contribute to heightened risk for the development of CVD [26], may further explain how CVD relates to long-term HRQOL. Nevertheless when accounting for likelihood of having a diagnosis for a postinjury PTSD or musculoskeletal condition and concurrent mental health symptoms, cardiovascular problems do not seem to drive or explain long-term links between injury type and HRQOL. Future research should examine the role of behavioral health factors in contributing to the development and maintenance of CVD, and their impact on the long-term prognosis and HRQOL of injured service members.

Although initially findings seems to contradict prior research suggesting EIs are associated with higher PTSD and other mental health symptoms [15, 18], this is likely a function of differences in the comparison group used across studies. As participants with EI were compared to those with TBI, this is not entirely surprising. A wealth of evidence suggests TBI is highly correlated with PTSD and other mental health concerns following injury [13, 21, 22]. It is important to note, however, that our study is not suggesting that EI is unrelated to PTSD symptoms or diagnosis; indeed, 35% of participants with an EI screened positive for current PTSD. However, EI is associated with lower likelihood of receiving a postinjury PTSD diagnosis relative to those with TBI. Given the comparable levels of PTSD symptoms endorsed by those with EI and TBI on the WWRP, this could indicate that PTSD is being underidentified among those with EI in the early stages following injury. Additional research is needed to examine this.

This study is not without limitations. First, although the statistical model accounted for temporality among the parameters and indirect effects, the study is correlational, and causation cannot be established. Further, the model was just-identified, restricting our ability to examine how well the model fit the data. Still, all modelled pathways were supported by prior research and theory. As previously mentioned, one potential limitation of the present study stems from our reliance on DoD medical records to determine whether participants had a documented postinjury PTSD, MSD, or CVD diagnosis. It remains possible that some visits were not accounted for if participants sought care outside the Military Health System, and it is currently unknown to what extent participants sought outside care. Another potential limitation is the lack of a noninjured comparison group. Although the findings of this study shed light on the experiences of service members with EI relative to those with TBI, these groups were mutually exclusive, and thus did not allow for the examination of comorbidity or polytrauma. Additional information could be gleaned from examining how this process unfolds relative to service members who were not injured or who sustained other injuries. Researchers examining mechanisms linking injury to long-term health outcomes should be mindful of and explicit about their respective comparison groups. Additionally, this study excluded some individuals who had both a documented EI and a probable TBI, potentially limiting its generalizability. Still, nearly 70% of the eligible WWRP sample was represented in this study, and EI and TBI remain characteristic injuries of the post-9/11 era, further supporting its overall representativeness.

## Conclusions

Service members who sustain EIs and TBIs experience myriad consequences that may place them at greater or lower risk for certain health complications. Importantly, the prevalence of probable PTSD was high among those with an EI or TBI relative to veteran populations and the general public. Relative to those with TBI, however, service members with an EI were more likely to have a postinjury musculoskeletal diagnosis and less likely to have a postinjury PTSD diagnosis. Further, higher likelihood for a musculoskeletal and lower likelihood for a PTSD diagnosis were associated with lower levels of PTSD symptoms, and higher subsequent physical and mental HRQOL. Moreover, injury type was shown to be indirectly related to long-term mental and physical HRQOL through both physical (i.e., likelihood of MSD diagnosis) and mental (i.e., likelihood of PTSD diagnosis and level of PTSD symptoms) health processes. Thus, optimized treatment plans and rehabilitation efforts are needed to address secondary mental and physical health conditions, and to promote readiness, health, and overall wellness of injured service members.

## Data Availability

The datasets generated and/or analyzed during the current study are not publicly available due to personally identifiable information regulations, but may be made available by the corresponding author on reasonable request and approval by the Naval Health Research Center Institutional Review Board.

## Abbreiviations

AIS: Abbreiviated injury scale
BCCI: Bias-corrected confidence interval
CVD: Cardiovascular disorders
DoD: Department of defense
EI: Extremity injury
EMED: Expeditionary medical encounter database
HRQOL: Health-related quality of life
ICD-9-CM: International classification of diseases, ninth revision, clinical modification
ICD-10-CM: International classification of diseases, tenth revision, clinical modification
ISS: Injury severity score
MCS: Mental health component score
MSD: Musculoskeletal disorder
PCL5: Posttraumatic stress disorder checklist for DSM-5
PCS: Physical health component score
PTSD: Posttraumatic stress disorder
SF-36: 36-Item short form survey
TBI: Traumatic brain injury
WLSMV: Weighted least square mean and variance adjusted estimation
WWRP: Wounded warrior recovery project
